# Multiclassification of Colorectal Polyps from Colonoscopy Images Using AI for Early Diagnosis

**DOI:** 10.3390/diagnostics15101285

**Published:** 2025-05-20

**Authors:** Jothiraj Selvaraj, Kishwar Sadaf, Shabnam Mohamed Aslam, Snekhalatha Umapathy

**Affiliations:** 1 Department of Biomedical Engineering, College of Engineering and Technology, SRM Institute of Science and Technology, Kattankulathur, Chengalpattu 603203, India; js0740@srmist.edu.in; 2 Department of Computer Science, College of Computer and Information Sciences, Majmaah University, Al Majmaah 11952, Saudi Arabia; 3 Department of Information Technology, College of Computer and Information Sciences, Majmaah University, Al Majmaah 11952, Saudi Arabia; s.aslam@mu.edu.sa

**Keywords:** colorectal cancer, colorectal polyp, CRP-ViT, multiclassification, colonoscopy images

## Abstract

**Background/Objectives:** Colorectal cancer (CRC) remains one of the leading causes of cancer-related mortality worldwide, emphasizing the critical need for the accurate classification of precancerous polyps. This research presents an extensive analysis of the multiclassification framework leveraging various deep learning (DL) architectures for the automated classification of colorectal polyps from colonoscopy images. **Methods:** The proposed methodology integrates real-time data for training and utilizes a publicly available dataset for testing, ensuring generalizability. The real-time images were cautiously annotated and verified by a panel of experts, including post-graduate medical doctors and gastroenterology specialists. The DL models were designed to categorize the preprocessed colonoscopy images into four clinically significant classes: hyperplastic, serrated, adenoma, and normal. A suite of state-of-the-art models, including VGG16, VGG19, ResNet50, DenseNet121, EfficientNetV2, InceptionNetV3, Vision Transformer (ViT), and the custom-developed CRP-ViT, were trained and rigorously evaluated for this task. **Results:** Notably, the CRP-ViT model exhibited superior capability in capturing intricate features, achieving an impressive accuracy of 97.28% during training and 96.02% during validation with real-time images. Furthermore, the model demonstrated remarkable performance during testing on the public dataset, attaining an accuracy of 95.69%. To facilitate real-time interaction and clinical applicability, a user-friendly interface was developed using Gradio, allowing healthcare professionals to upload colonoscopy images and receive instant classification results. **Conclusions:** The CRP-ViT model effectively predicts and categorizes colonoscopy images into clinically relevant classes, aiding gastroenterologists in decision-making. This study highlights the potential of integrating AI-driven models into routine clinical practice to improve colorectal cancer screening outcomes and reduce diagnostic variability.

## 1. Introduction

**Figure 1:**
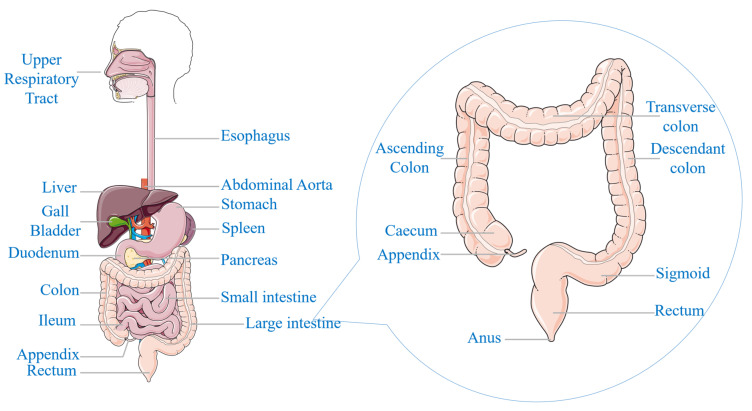
Anatomical representation of the human digestive system.

The cells within the human body normally undergo a controlled cycle of proliferation and senescence, ensuring tissue homeostasis [1,2]. However, in the development of cancer, this process is disrupted, resulting in the uncontrolled proliferation of abnormal cells [3,4]. These aberrant cells can also invade surrounding tissues and can metastasize to distant organs, compromising normal physiological functions [5,6]. Colorectal cancer (CRC) is a malignant condition that originates in the epithelial tissues of the large intestine (colon) or the final segment of the digestive tract (rectum) [7,8,9], as shown in Figure 1. CRC often originates as a benign growth known as a colorectal polyp (CRP), which can develop into an invasive cancer [10]. While the majority of polyps are benign, certain types have the potential to undergo malignant transformation over an extended period [11]. The probability of a polyp progressing to cancer is determined by its histological classification, encompassing the structural and cellular attributes of the polyp [12,13].

Different types of CRPs include the following:

- Hyperplastic and inflammatory polyps: These types are more frequently encountered and are generally considered non-precancerous. However, individuals with large hyperplastic polyps (>1 cm) may require more frequent CRC screening through colonoscopy, as a precautionary measure [14,15].
- Serrated polyps: Sessile serrated polyps (SSPs) and traditional serrated adenomas (TSAs) are two common premalignant lesions that share an increased risk of progression to CRC. SSPs are characterized by their relatively flat or slightly elevated morphology and indistinct borders. Conversely, TSAs often exhibit a more pedunculated or sessile growth pattern and a more pronounced adenomatous behaviour, distinguishing them from SSPs [16,17].
- Adenomatous polyps (adenomas): These polyps possess the potential to evolve into cancer and thus are regarded as precancerous. Adenomas are further classified into subtypes: tubular, villous, and tubulovillous [18]. Among these, tubular adenomas are the most prevalent, while villous adenomas, though less common, carry a higher risk of malignancy [19].

**Figure 2:**
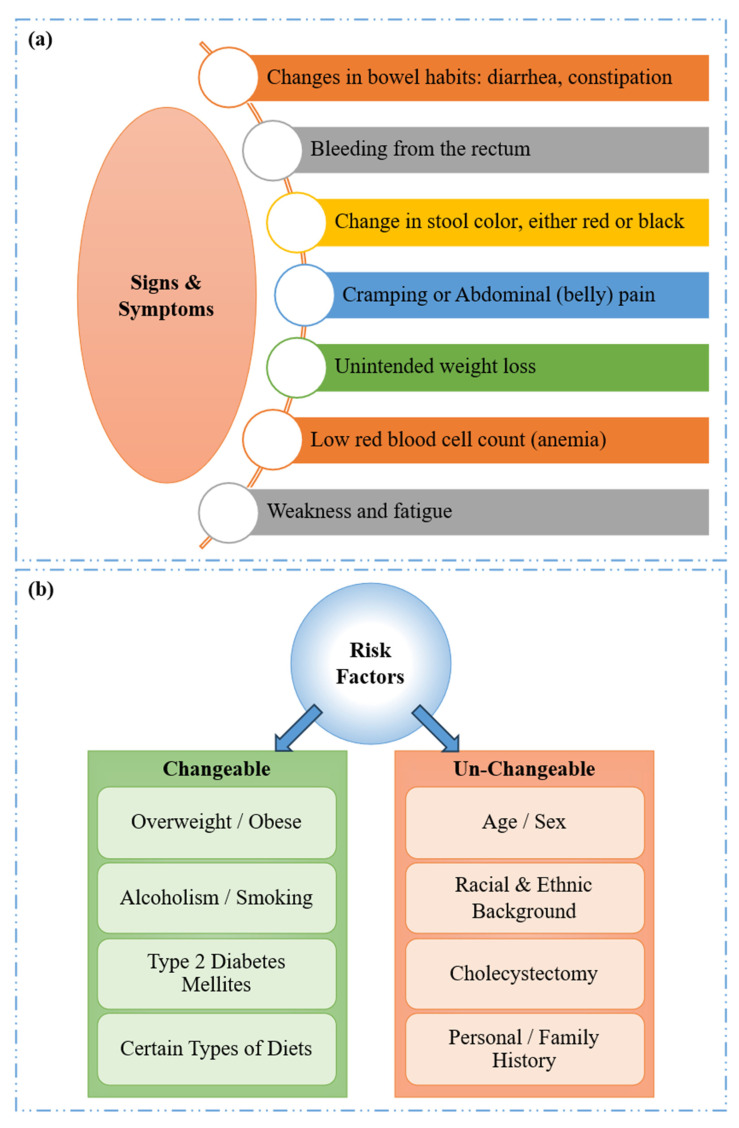
CRC: (**a**) Signs and symptoms and (**b**) risk factors.

The key signs and symptoms associated with CRC/CRP [9,20] are persistent alterations in bowel habits and rectal bleeding as represented in Figure 2a. A notable symptom is tenesmus, where patients experience a continuous sensation of incomplete bowel evacuation, unrelieved by defecation. Other associated symptoms include lower abdominal cramps, unexplained fatigue, generalized weakness, and significant unintentional weight loss [21].

The key risk factors associated with CRC may be either changeable or un-changeable. As illustrated in Figure 2b, changeable factors, which can be altered through lifestyle interventions or environmental changes, include obesity, excessive alcohol consumption, type 2 diabetes, smoking, and specific dietary patterns. These factors are manageable and can potentially reduce the risk when addressed. On the other hand, the un-changeable risk factors, which cannot be altered, include age, race/ethnicity, sex, a history of cholecystectomy (gallbladder removal), and a personal or family history of CRC, CRP, or inflammatory bowel disease (IBD) [22,23].

**Figure 3:**
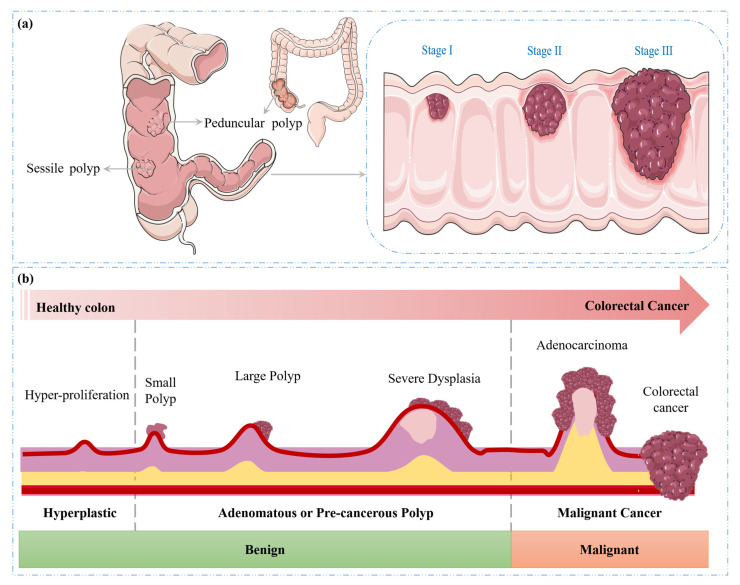
Progression of CRC. (**a**) Types of polyps and (**b**) transition from benign to malignant.

CRC can lead to significant complications as the malignancy progresses, as it can invade adjacent tissue layers, potentially penetrating the muscularis propria and serosa. This invasive growth can facilitate the dissemination of cancer cells via the lymphatic and vascular systems, leading to the formation of metastases in regional lymph nodes and distant organs [24,25]. The stage of CRC, a critical prognostic factor, is determined by the extent of tumour invasion and the presence or absence of distant metastases, reflecting the local and systemic spread of the disease [26]. The progression of colorectal cancer (CRC) from normal colonic tissue to malignant cancer is illustrated in Figure 3a,b.

**Figure 4:**
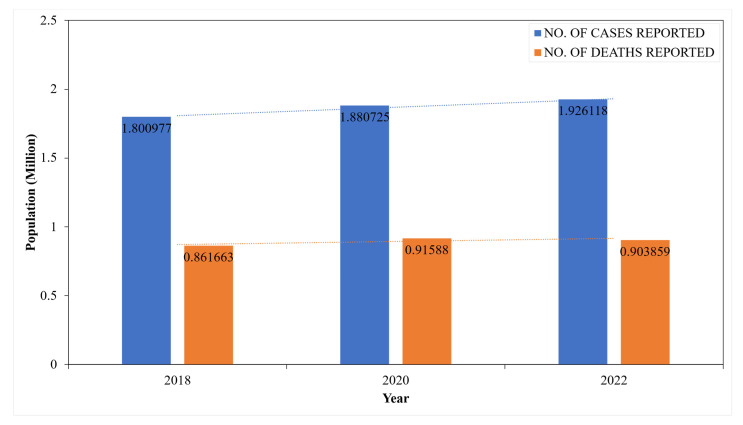
CRC statistics: WHO (GLOBOCON).

The statistics of CRC are represented as a bar chart in Figure 4. The CRC incidence (blue bar) and death rates (orange bar) are reported from 2018 to 2022 [27,28,29]. The trend lines highlight the gradual progression from 1.80 to 1.93 million in incidence and 0.86 to 0.90 million death rates over the years. According to statistics from GLOBOCAN, CRC ranks as the third most globally diagnosed cancer and the second leading cause of cancer-related mortality [7].

**Figure 5:**
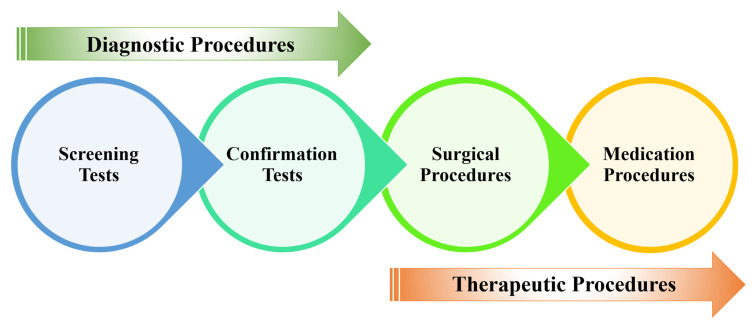
CRC D&T pathways.

Figure 5 presents a schematic overview of the diagnostic and therapeutic (D&T) pathways for CRC. Screening procedures serve as an initial assessment to identify potential cases of CRC prior to the onset of overt symptoms [30]. Upon the detection of anomalies in screening tests, confirmatory diagnostic evaluations (biopsy) are conducted to establish a definitive diagnosis of CRC. Subsequent to diagnosis, surgical intervention is employed to excise the primary tumour [31]. Postoperative management may involve adjuvant therapies, including chemotherapy, immunotherapy, or other pharmacological interventions to manage the disease or to prevent recurrence [32].

**Figure 6:**
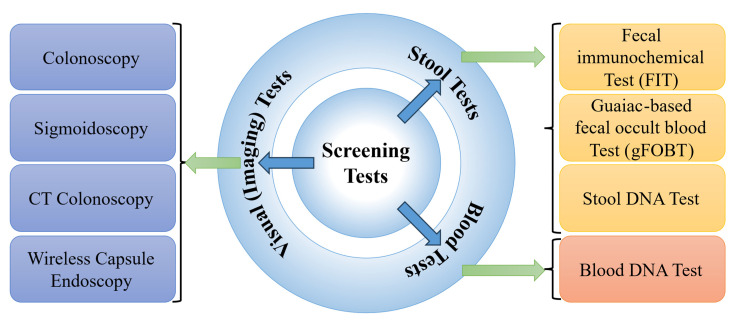
Types of CRC screening methods.

The three primary techniques for CRC screening, including blood-, stool-, and image-based (visual) examinations [33], are presented in Figure 6. Blood-based assays analyze a patient’s blood for molecular or protein markers indicative of colorectal malignancies. Stool-based non-invasive assays evaluate fecal samples for the potential biomarkers of CRC, such as occult blood or abnormal DNA [34]. While these tests offer a less invasive screening method compared to other options, they generally require more frequent repetition to maintain efficacy [35]. Visual examinations involve the direct visualization of the colon and rectum to identify structural abnormalities, such as polyps or tumours [36]. This can be achieved either via endoscopy (e.g., colonoscopy, sigmoidoscopy, wireless capsule endoscopy (WCE)), equipped with a light source and video camera, or through radiographic techniques like computed tomography (CT) colonography [37].

**Figure 7:**
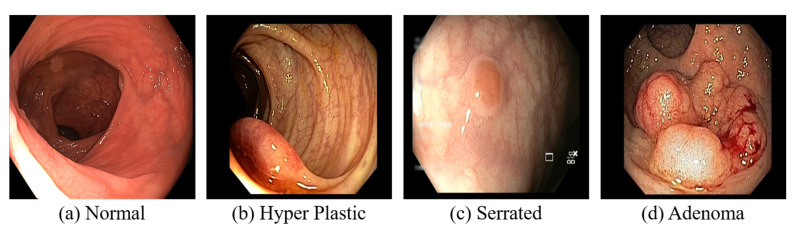
Colonoscopy images.

A CT colonography, where the CT of the colon and rectum is performed, is less sensitive in detecting small polyps (<5 mm in diameter) [38]. Despite the relatively low radiation exposure from CT colonography, it remains a consideration, and since the procedure is non-therapeutic, the detected polyps cannot be removed, potentially necessitating a follow-up colonoscopy [39]. A colonoscopy examination involves a comprehensive visual assessment of the colon and rectum. A colonoscope, a slender, flexible tube equipped with a light source and a miniature camera, is inserted through the anus and gently advanced into the colon. This procedure allows for the direct visualization of the entire colonic lumen, facilitating the identification and evaluation of potential pathological lesions. If necessary, specialized instruments can be passed through the colonoscope to obtain tissue biopsies or perform an endoscopic resection of suspicious abnormalities, such as colonic polyps [40]. The normal and CRC are visually depicted in Figure 7. Sigmoidoscopy, similar to colonoscopy, facilitates examining only the lower third of the colon and the entire rectum, which may increase the likelihood of missing lesions located in the proximal colon [41]. In WCE, a camera-equipped capsule is ingested by the patient, enabling the transmission of images as it traverses the GI tract [42]. The capsule’s battery life is often insufficient to fully capture images of the colon and rectum, especially in patients with slow GI transit time.

In a clinical setting, for visual examination, colonoscopy is preferred rather than other imaging techniques due to its advantages compared to other procedures [43,44]. The visual examination of the colonoscopy images by the physician is tedious and time-consuming [45]. Due to the subjective nature of visual interpretation, clinicians may inadvertently overlook polyps or lesions, particularly those of diminutive size or flat morphology [46].

Traditional colonoscopy, while effective, can be limited by human factors such as fatigue or variability in interpretation. AI algorithms can automatically analyze colonoscopy images with high precision, identifying subtle patterns and abnormalities that might be missed by human eyes [47,48]. This technological integration aids in early detection, improves diagnostic accuracy, and reduces the risk of missed lesions, ultimately leading to better patient outcomes [49]. Machine learning (ML) algorithms, employing manual feature engineering, have exhibited significant proficiency in the detection of CRPs within the colonoscopy images. Contemporary research efforts are increasingly focused on deep learning (DL) architectures, notably convolutional neural networks (CNNs), eliminating the need for manual feature extraction. The neural network’s intrinsic capacity to learn and extract relevant features directly from the input colonic images has demonstrated promising results in both segmenting and classifying the CRP [50].

### 1.1. Literature Review

Colorectal polyps can be classified based on their characteristics into several categories, including hyperplastic, adenomatous, and serrated polyps. The classification of these polyps is crucial for determining the risk of colorectal cancer, as certain types, particularly neoplastic polyps (adenomatous and serrated), have a higher potential for malignancy. In the endoscopic evaluation of colorectal polyps, classification systems such as Kudo [51], NICE [52], Paris [53], and Sano [54] have a significant role in guiding clinical decisions. The Kudo pit pattern classification focuses on the mucosal surface architecture, using magnifying chromoendoscopy to distinguish between non-neoplastic and neoplastic lesions based on pit patterns I to V, where advanced irregular patterns suggest invasive carcinoma. Complementing this, the narrow-band imaging international colorectal endoscopic (NICE) classification leverages image-enhanced endoscopy to categorize lesions into types 1, 2, and 3 based on colour, vascular pattern, and surface structure. The Paris classification, on the other hand, provides a morphological framework to describe superficial neoplastic lesions, categorizing them into protruding, non-protruding, and excavated types, which is crucial for assessing the risk of submucosal invasion. Meanwhile, the Sano capillary pattern classification emphasizes microvascular architecture under magnification with narrow-band imaging, particularly highlighting irregular or sparse vascular patterns that are indicative of deeper invasion. Integrating these complementary classifications enhances the accuracy of endoscopic assessment, enabling detection and risk stratification for colorectal neoplasia.

This literature review synthesizes findings from multiple studies investigating the effectiveness of AI techniques in enhancing the detection and classification of colorectal polyps. Notably, Komeda et al. [55]. developed a CNN-based computer-aided diagnosis (CAD) system, presenting promising preliminary results that underscore the potential of AI in colon polyp detection. Their pilot study involved a retrospective analysis of colonoscopy video frames to identify polyps that might otherwise be missed by human observers. The researchers specifically focused on classifying colon images into adenomatous and non-adenomatous polyps, providing an early indication of AI’s capability to support diagnostic decision-making in clinical practice.

A systematic review by Sanchez-Peralta et al. [56] analyzed various AI methods for polyp detection, highlighting the advantages and disadvantages of different approaches. The authors insisted on the necessity for a common validation framework that includes a large, annotated database, which is crucial for standardizing results and facilitating comparisons across studies. Many studies have shown that the size and histological type of polyps significantly influence cancer risk, with larger and more complex polyps being associated with a higher likelihood of malignancy [12,57]. Itoh et al. [58] developed an automated binary classification based on the size of the polyp. Saad et al. [59] advanced this approach by implementing a multiclass classification system using the PICCOLO dataset, categorizing polyps into six distinct classes according to the Paris classification. Grosu et al. [60] proposed an ML-based classification of benign and pre-malignant polyps. Sharma et al. [61] developed a two-stage binary classification model for identifying cancerous polyps using an ensemble-based CNN model. Barua et al. [62] identified neoplastic polyps through binary classification methods using CAD.

In contrast to the more common binary classification of polyp presence, the literature contains limited studies on multiclassification. The application of AI for the multiclass classification of CRPs from colonoscopy images has gained substantial attention in recent years, primarily due to its potential to facilitate early diagnosis and improve patient outcomes. Bora et al. [63] proposed a novel approach to quantify shape, texture, and colour features for detecting the stages of dysplasia in polyps using a fuzzy entropy-based feature selection method, achieving an accuracy of 95.24% in the generated dataset and 95.72% in the public dataset. Krenzer et al. [64] focused on the development of two automated classification systems for polyps in the field of gastroenterology: The first system, based on the Paris classification, which categorizes polyps based on their shape, and the second system, based on the NICE classification, which categorizes polyps based on their texture and surface patterns [65].

The study by Wang et al. [66] utilized a deep learning approach for the automatic detection of colonic polyps using CNNs with global average pooling, achieving a high classification accuracy of 98% and reducing model parameters. Carvalho et al. [67] proposed a DL model to classify polyp features according to the NICE classification, achieving an accuracy of over 92% on internal datasets and exceeding 88% on a public dataset, demonstrating its potential to enhance diagnostic decision-making in CRC diagnosis. The present work distinguishes itself from the existing literature by advancing beyond conventional binary and limited multiclass classification systems that describe the severity of CRPs.

A customized four-class classification model that includes hyperplastic, serrated, adenoma, and normal categories is proposed in this research. Our approach also integrates the AI model into an interactive, web-based Gradio interface, enabling real-time, user-friendly deployment for clinical practitioners.

### 1.2. Contributions

Aim and Objectives: The aim of this investigation is to develop an AI-dependent multiclassification model for the early diagnosis of CRPs from colonoscopy images, enhancing clinical accuracy and aiding in the timely detection and prevention of CRC. The objectives of this study encompass the collection and preprocessing of colonoscopy image datasets for polyp classification, the design and implementation of deep learning algorithms for the multiclass classification of various types of colorectal polyps, and the evaluation of the proposed AI model’s performance using standard metrics. Additionally, the research involves comparing the developed model with existing state-of-the-art techniques in polyp classification and validating the model’s effectiveness to ensure clinical applicability.

Based on the objectives, the author contributions are as follows:

- Dataset collection and preprocessing: A comprehensive colonoscopy image dataset including real-time data is curated and preprocessed to enhance the quality of input data for effective CRP classification. Additionally, ethical clearance is obtained to ensure the responsible collection and use of real-time images in compliance with regulatory guidelines.
- AI-based deep learning model development and performance evaluation: A customized DL-based multiclassification model is designed and implemented to accurately classify different types of colorectal polyps, aiding in the early diagnosis of CRC. The proposed model’s performance is assessed using standard evaluation metrics.
- Comparative analysis: The developed AI model is compared with existing state-of-the-art methods for polyp classification to demonstrate its superiority and effectiveness in clinical diagnosis.
- Interactive interface deployment using Gradio: To enhance usability and clinical translation, a Gradio-based interface is developed, enabling users to upload colonoscopy images and receive immediate visual feedback on the predicted type of polyp. This interactive feature aids clinicians in real-time decision-making during diagnostic procedures.

This research article is structured to provide a comprehensive exploration of the study. It begins with an introduction, outlining the context and background, followed by an in-depth literature review and a clear statement of the contributions made by the study. The Materials and Methods (Section 2) details the proposed workflow, including data acquisition, preprocessing, and the implementation of deep learning classifiers, along with the integration of a real-time diagnostic interface using Gradio-5.29.0. The Results and Discussion (Section 3) presents and analyzes the findings, providing insights into the model’s performance and potential future directions. Finally, the article concludes (Section 4) with a summary of the research outcomes.

## 2. Materials and Methods

### 2.1. Proposed Workflow

**Figure 8:**
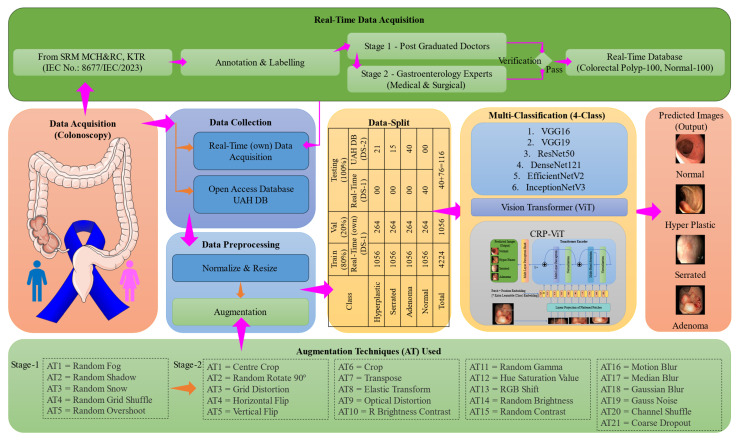
Proposed workflow.

Figure 8 illustrates the workflow of the proposed research methodology. The entire experimental framework was implemented using the Python-3.10 programming language. In this study, two datasets were utilized. The real-time dataset, following data augmentation, was employed for training and validation. To evaluate the generalizability of the architecture, non-augmented images from both the publicly available dataset and the real-time dataset were used exclusively for testing. As illustrated in Figure 8, initial experiments were conducted using six conventional CNN architectures, including VGG16 [68], VGG19 [68], ResNet50 [69], DenseNet121 [70], EfficientNetV2 [71], and InceptionNetV3 [72]. VGG16 and VGG19 are known for their uniform layer design and lower computation complexity. ResNet50 introduces residual connections that enable the training of deeper networks without the vanishing gradient problem, leading to improved accuracy and convergence. DenseNet121 enhances features through dense connectivity between layers, resulting in reduced parameters and strengthened gradient flow. InceptionNetV3 incorporates multi-scale processing within each module, enabling the efficient handling of visual information. EfficientNetV2 offers state-of-the-art performance by scaling depth, width, and resolution in a compound manner, achieving high accuracy with optimized computational efficiency. The inclusion of these diverse models allows for a robust comparative evaluation of their capabilities in detecting and classifying colorectal polyps from colonoscopy images.

Additionally, Transformer-based models, specifically the Vision Transformer (ViT) [73], were incorporated. Building upon the performance of these baseline models, a hybrid architecture combining ResNet50 and ViT was developed, herein referred to as CRP-ViT. Furthermore, the Gradio library [74] was utilized to develop the graphical user interface (GUI) for facilitating class prediction.

### 2.2. Data Acquisition and Data Collection

In this study, two distinct datasets (DSs) were utilized to conduct the investigations: a real-time dataset, denoted as DS-1, and a publicly available/open access (OA) (UAH) dataset [16], referred to as DS-2. DS-1 comprises data collected from the SRM Medical College Hospital and Research Centre, Kattankulathur, India. The research protocol for DS-1 was reviewed and approved by the Institutional Ethics Committee (IEC)—Human Studies of SRM Medical College Hospital and Research Centre under approval number 8677/IEC/2023. The study design adhered to ethical guidelines, ensuring that all procedures, including participant recruitment, informed consent, data collection, and analysis, were conducted, with strict adherence to ethical standards to protect participants’ rights and welfare. Each video within DS-1 is annotated with images obtained from histopathological analysis, complemented by an assessment performed by two expert evaluators and two novice observers referring to NICE and Sano classification. The annotation and labelling methodology, along with additional dataset details, are comprehensively illustrated in Figure 8.

DS-2 [16], on the other hand, was sourced from an open access, publicly available database. Similarly to DS-1, each video in DS-2 is accompanied by ground truth derived from histopathology and expert assessments. However, DS-2 features a more diverse panel of evaluators, consisting of four expert reviewers and three beginners, ensuring a balanced representation of varying levels of expertise. The multi-tiered annotation approach enhances the robustness of the dataset, facilitating a more comprehensive validation of the region of interest in the colonoscopy images.

Table 1 presents an overview of the datasets, including DS-1 and DS-2, utilized for CRP detection through colonoscopy image analysis. DS-1, collected from 71 participants, comprising 100 polyp frames and 100 normal frames, was captured at a high resolution of 1920 × 1080 pixels. DS-2 [16], consisting of 76 polyp frames from 76 participants, with no normal images available, has a resolution of 768 × 576 pixels. The distribution of images under each class of polyp is elaborated in Table 2. It is notable that the distribution is uneven across each class considered in this investigation.

### 2.3. Image Preprocessing and Data Split

In this study, three essential preprocessing steps, including resizing, data normalization, and data augmentation, were implemented to improve the performance of the DL models [7]. To ensure uniformity across datasets DS-1 and DS-2, all the frames were resized to 512 × 512. Additionally, following the resizing of images, data normalization was performed concurrently to enhance the consistency of the images. DS-1, excluding 40 normal images, was considered for training and validation. DS-2, along with 40 normal images excluded from DS-1, was utilized for testing the DL model. Data augmentation on DS-1 was conducted in two stages: Stage 1 and Stage 2. The primary objective of Stage 1 augmentation was to balance the distribution of polyp classes, while Stage 2 augmentation aimed to increase the dataset size for more effective training and validation.

In Stage 1 augmentation, a balanced dataset was achieved, with each class containing 60 images. As already 60 hyperplastic images were available, augmentation techniques were applied to balance the remaining polyp classes. The serrated class was augmented using one technique, while the adenoma class underwent five augmentation techniques. Among the 100 normal images of DS-1, 60 were considered without any augmentation. In the Stage 2 augmentation, all the images considered for training and validation, totalling 60 images per class across four classes, were augmented using 21 techniques. As a result, the dataset for training and validation following the Stage 2 augmentation was 5280 images, as presented in Table 3. The utilized augmentation techniques are clearly illustrated in Figure 8. Following Stage 2 augmentation, 80% of the data (4224) was used for training, and the rest, 20% of the frames (1056), were used for validation. To assess the generalizability of the proposed system, 116 images, including images from DS-1 (40 normal images) and DS-2 (76 images), were used as detailed in Table 4.

### 2.4. Deep Learning Classifier

**Figure 9:**
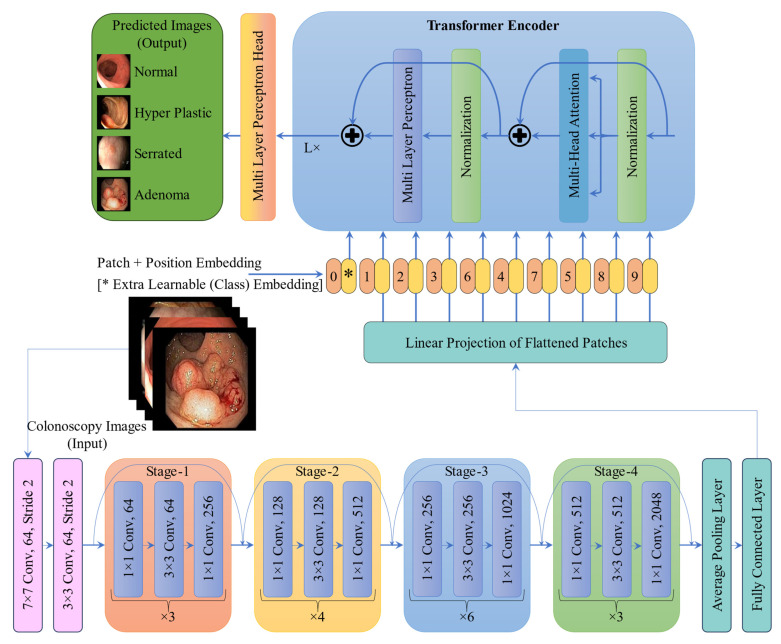
Architecture of CRP-ViT (multi-class).

The CRP-ViT architecture, illustrated in Figure 9, originally developed for the binary classification of polyp presence in the previous study [7], was used in the present work as well. However, instead of the sigmoid function, the final layer was replaced with a SoftMax activation to better align with the multiclassification task. The CRP-ViT integrates ResNet50 with ViT encoders to enhance the classification of colonoscopy images. Initially, high-resolution images are passed through a series of convolutional layers, beginning with a 7 × 7 convolution followed by 3 × 3 convolutions, to extract low-level spatial features. These are further processed through a ResNet50 feature extractor consisting of four sequential stages, each containing multiple stages of residual blocks with 1 × 1 and 3 × 3 convolutions that progressively increase the number of channels and capture rich hierarchical representations. The resulting feature maps are then divided into non-overlapping patches and linearly projected into a suitable embedding space to serve as input tokens for the ViT encoder. The ViT encoder, composed of multiple stacked layers of multi-head self-attention, layer normalization, and multi-layer perceptrons (MLPs), enables global contextual modelling across the entire image. The encoded features are then passed through a final MLP head for classification. This hybrid architecture effectively combines the local feature extraction capabilities of CNNs with the global modelling power of Transformers, making it well suited for the accurate detection and classification of CRPs.

### 2.5. Real-Time Diagnostic Interface Integration Using Gradio

The Gradio, a Python library [74], allows the creation of an interactive web-based interface without any complex skills. In this research, Gradio is utilized to build an intuitive interface through which clinicians can upload colonoscopy images and immediately view the predicted type of colorectal polyp generated by the DL model. This real-time interaction significantly enhances the usability of the system, supporting clinicians in making timely and informed decisions during diagnostic procedures. Moreover, Gradio facilitates the rapid prototyping and sharing of the DL model considered in this study by generating deployable web applications that can be accessed locally or shared via public links. By integrating Gradio, the proposed framework bridges the gap between advanced DL algorithms and practical clinical application, promoting accessibility, transparency, and ease of use in clinical settings.

## 3. Results and Discussion

The performance metrics of conventional DL models are summarized in Table 5, Table 6, Table 7, Table 8, Table 9 and Table 10. The performance was assessed across four individual classes: Class 0—normal, Class 1—hyperplastic, Class 2—serrated, and Class 3—adenoma. The evaluation was conducted using three distinct optimizers, with each model trained for 50 epochs. The number of epochs in which the model converged varied depending on the model and the optimizer used. The learning rate of the classifier was set to 0.0001 based on insights from prior work on binary classification.

ResNet50, when optimized with ADAM, exhibited superior performance with the highest overall training accuracy of 89.2% and validation accuracy of 88.07%, indicating its capability to effectively capture complex features and handle multiclassification. VGG19, with ADAM, also demonstrated competitive performance, while VGG16 showed a moderate performance. The ADAM optimizer demonstrated superior convergence and stability across all architectures, while RMSprop optimization showed balanced performance, slightly inferior when compared to ADAM. Overall, the findings emphasize that the choice of optimizer plays a crucial role in enhancing the model’s performance. The ADAM has emerged as the most effective optimizer for improving classification accuracy and stability across different classes in this investigation.

DenseNet121, EfficientNetV2, and InceptionNetV3 demonstrated competitive classification performance, compared with ResNet50, particularly when trained using the ADAM optimizers. Among the evaluated architectures, ResNet50 and EfficientNetV2 yielded closely comparable performance metrics, exhibiting minimal variance across training and validation phases. However, ResNet50 consistently outperformed EfficientNetV2, achieving a training accuracy of 89.2% and a validation accuracy of 88.07%, in contrast to EfficientNetV2’s 88.66% and 87.69%, respectively. A comprehensive breakdown of model-specific performance metrics is detailed in Table 7 and Table 9. Based on this consistent performance advantage, the ResNet50 architecture was selected for further experimentation in subsequent phases of the study.

The Transformer-based model, ViT, was analyzed across the three optimizers and is presented in Table 11. The ViT optimized with ADAM performed well compared to the other optimizers considered in this investigation, with an overall accuracy of 92.38% and 95.36% during training and validation. The RMSprop achieved performance in close association with the ADAM, while the SGD showed the lowest performance, with slower convergence and a limited ability to handle complex data patterns.

The performance achieved by CRP-ViT is tabulated in Table 12. When evaluating the performance of CRP-ViT, the ADAM optimizer achieved the highest metrics, with a training accuracy of 97.28% and a validation accuracy of 96.02%. Across all scenarios, ADAM proved to be the most effective optimizer, owing to its adaptive learning strategy and superior convergence capabilities. Furthermore, when comparing the performance of all models, CRP-ViT exhibited exceptional performance, outperforming its counterparts.

In the comparative evaluation of deep learning architectures for colorectal image classification, the CRP-ViT model demonstrated superior performance across the metrics, outperforming traditional CNN and Transformer models, reflecting its robust feature extraction and class discrimination capabilities. The ViT model also exhibited commendable performance, especially with the ADAM optimizer, surpassing traditional models, thus highlighting the efficacy of Transformer-based architectures in capturing complex patterns within colorectal images. While ResNet50, with its deep residual connections, offered improved accuracy over the VGG architectures, it still lagged behind the ViT and CRP-ViT. VGG16 and VGG19, despite their historical significance and simplicity, showed relatively lower performance, likely due to their limited capacity in modelling intricate spatial relationships compared to ViT and CRP-ViT. Overall, the integration of convolutional mechanisms within the CRP-ViT model substantially enhanced classification efficacy, making it a promising approach for advancing automated colorectal cancer screening.

The results of the K-fold cross-validation, which assessed the fold-wise performance and overall average performance of the CRP-ViT model, are presented in Table 13. A five-fold cross-validation across four distinct classes (Class 0, Class 1, Class 2, and Class 3) was implemented. The performance of the five-fold cross-validation was in close concordance with the 80:20 split. Furthermore, the five-fold cross-validation results demonstrate minimal performance variance across folds, as evidenced by the low standard deviation values across the key metric, accuracy. This suggests that the CRP-ViT maintains stable predictive performance irrespective of data partitioning, and it reinforces the model’s potential applicability in clinical settings.

The difference between the performance of the 80:20 split and five-fold cross validation is provided in Table 14. The marginal difference observed highlights the model’s ability to generalize effectively across different data splits, demonstrating that the model is not biased toward any specific split. The ablation study conducted on CRP-ViT is provided in Table 15. The analysis of ablation studies on different stages of the CRP-ViT architecture reveals significant variations in classification accuracy. Among the individual stages, ablation on Stage 3 of ResNet50 yields an accuracy of 92.78%, and Stage 2 of ResNet50 ablation follows closely with an accuracy of 91.71%. The Stage 4 and Stage 1 of ResNet50 ablations result in accuracies of 90.74% and 90.18%, respectively. Notably, ablation across all stages from Stage 1 to Stage 4 of ResNet50 drastically reduces the accuracy to 67.06%, highlighting the collective importance of these stages for effective feature extraction and learning. Ablation on the fully connected network (FCN) impacts the performance as well, dropping the accuracy to 87.85%. In comparison, the baseline model without any ablation achieves the highest accuracy of 97.28%, emphasizing the significance of maintaining the integrity of the entire architecture for optimal performance.

**Figure 10:**
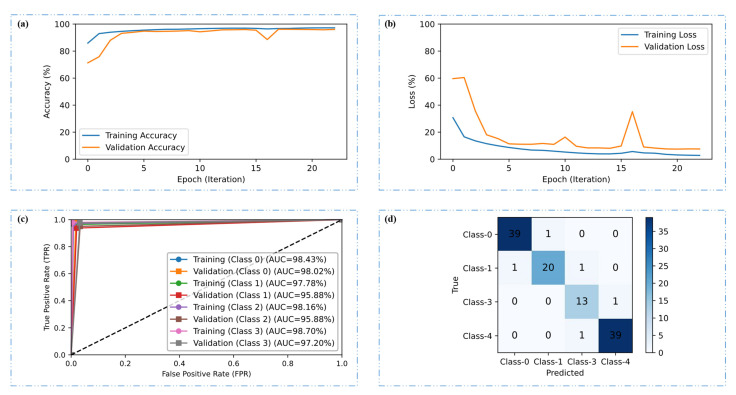
CRP-ViT (multi-class) classification results. (**a**) Accuracy plot, (**b**) loss plot, (**c**) ROC plot, and (**d**) confusion matrix for test results.

Figure 10a,b represent the training and validation performance of the CRP-ViT model across the epochs, highlighting both accuracy and loss trends. In the accuracy plot, the model demonstrates a steady increase in accuracy over the epochs, with both training and validation accuracy converging at the epoch of 23, indicating effective learning. In the loss plot, there is a clear downward trend in both training and validation loss values over time. The training loss shows a smooth and continuous decline, reflecting effective optimization. However, the validation loss, while decreasing, exhibits occasional spikes and stabilizes toward the latter epochs. The ROC curves for both the training and validation phases across the four distinct classes considered in this investigation, highlighting the model’s strong classification capability with consistently high AUC values, are presented in Figure 10c. Overall, the close alignment of the ROC curves between the training and validation sets, coupled with the high AUC scores across all classes, confirms the performance of CRP-ViT in multiclass classification, with minimal overfitting and strong predictive accuracy. The confusion matrix of 116 test images is provided in Figure 10d. Out of the 116 test images, the CRP-ViT has classified 111 images correctly, highlighting the model’s strong ability to distinguish between classes, with high true positive rates and minimal misclassifications.

**Figure 11:**
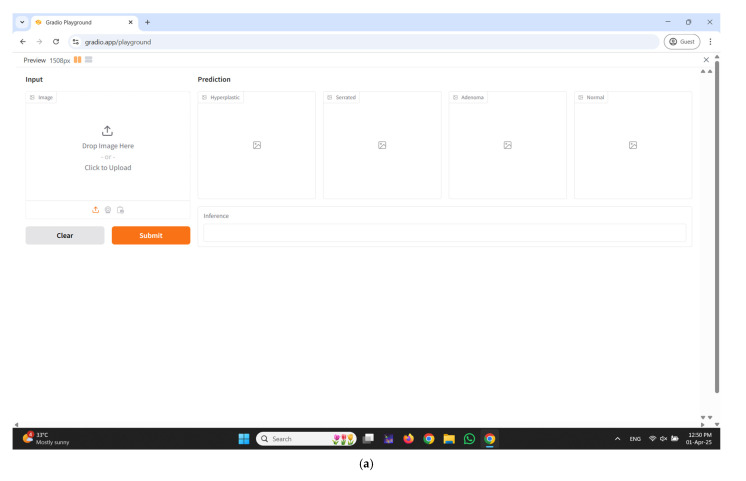

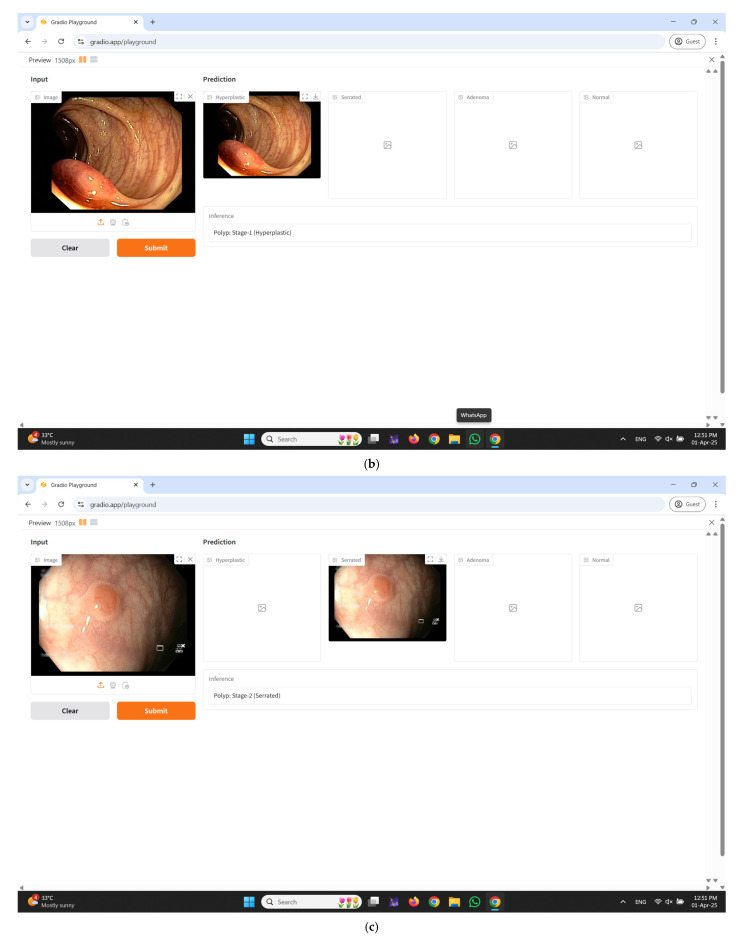

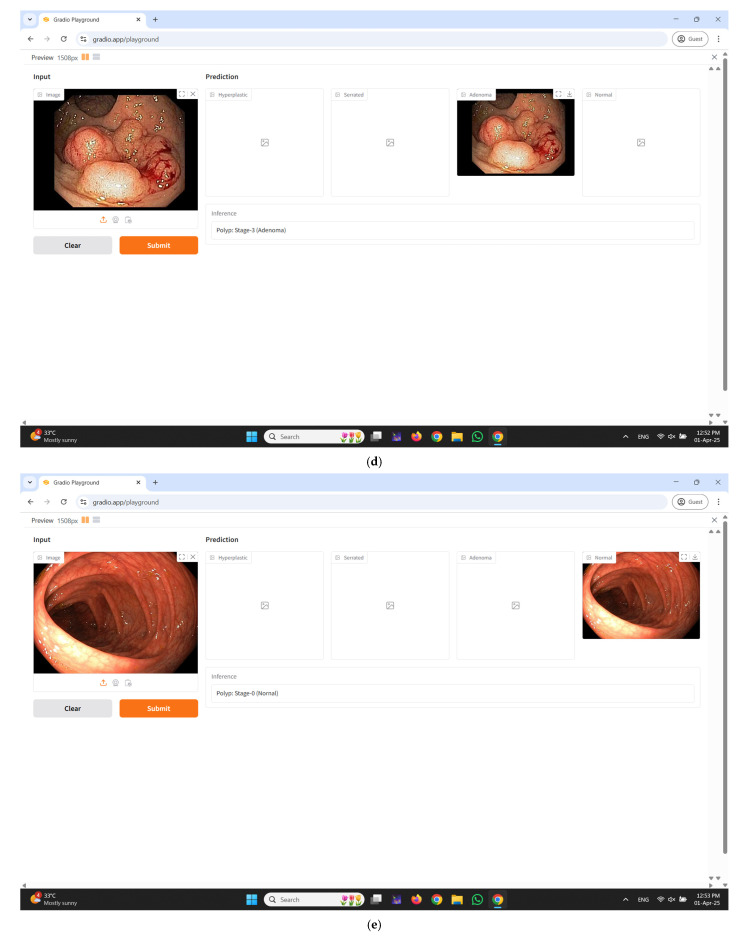
Developed user interface using Gradio. (**a**) Home Page; (**b**) predicted result: hyperplastic; (**c**) predicted result: serrated; (**d**) predicted result: adenoma; (**e**) predicted result: normal.

An interactive web-based interface developed using Gradio for real-time colorectal polyp classification is illustrated in Figure 11a–e. The input panel enables healthcare professionals to upload colonoscopy images either by dragging and dropping or selecting files manually from their local system. Upon submission, the system processes the input image through the proposed CRP-ViT model and displays the classification outcomes in the prediction section. The output is systematically categorized into four clinically significant classes: hyperplastic, serrated, adenoma, and normal, with each category distinctly displayed in separate panels to facilitate clear interpretation. Additionally, an inference text box is integrated to present the final decision, indicating the most probable class predicted by the model, thereby enhancing diagnostic clarity. The interface incorporates essential functional controls, such as the Clear button to reset the input selection and the Submit button to initiate the prediction process.

Most of the previous studies have primarily focused on binary classification approaches. Although the UAH DB dataset has been employed in the existing literature, its application has been limited to binary classification tasks. To date, no published studies have demonstrated the use of this dataset for multiclass classification, and therefore, a direct comparison with prior work is not feasible. For a fair performance evaluation, results from different datasets and studies cannot be directly compared. Furthermore, to the best of our knowledge, no publicly available dataset currently supports multiclass classification of this nature. Consequently, we utilized a self-collected dataset for model training and validation, while a publicly available dataset was employed solely for testing purposes.

Due to the absence of multiclass classification studies on CRPs using the same dataset employed in this research, a fair comparison with the existing literature was not feasible. Therefore, the proposed CRP-ViT model was benchmarked against six conventional CNN-based state-of-the-art models within this study. This internal evaluation strategy ensures a consistent and reliable comparison under uniform experimental conditions.

Limitations and future work: In the current study, the dataset comprised 4224 images for training, 1056 images for validation, and 116 images for testing. While the model demonstrated promising performance, the limited size and scope of the dataset constrain its ability to generalize across broader clinical environments. Future work will prioritize the expansion of the dataset by incorporating larger, multi-centre, and heterogeneous image collections to enhance the robustness and external validity of the model. Additionally, the integration of quantum computing techniques will be explored to accelerate and improve the precision of polyp classification tasks. Furthermore, the current model, which operates on static colonoscopy images, will be extended to process continuous colonoscopy video streams to enable real-time polyp detection and classification, thereby enhancing its applicability in live clinical settings and supporting endoscopists during procedures.

## 4. Conclusions

This study presents a comprehensive DL framework for the multiclass classification of colorectal polyps, aiming to enhance the detection and diagnosis of colorectal cancer (CRC). By leveraging real-time clinical data alongside publicly available datasets, the proposed approach ensures both robustness and generalizability. The custom-developed CRP-ViT model demonstrated exceptional performance, surpassing established architectures, such as VGG16, VGG19, ResNet50, DenseNet121, EfficientNetV2, InceptionNetV3, and ViT, by effectively capturing intricate patterns within colonoscopy images. The integration of the CRP-ViT model with an accessible Gradio-based interface bridges the gap between complex AI solutions and frontline clinical practice, enabling timely, accurate assessments even in resource-constrained settings. By reducing diagnostic variability and supporting medical professionals in real-time decision-making, the proposed framework contributes to more equitable healthcare delivery. Ultimately, this research paves the way for the widespread adoption of AI-assisted screening tools, fostering earlier interventions, improving patient survival rates, and alleviating the broader public health burden associated with colorectal cancer.

## Figures and Tables

**Table 1:** Dataset used in the proposed study.

Database	Participants	Still Frame		Resolution	Type
		Polyp	Normal		
Real-Time (own) (DS-1)	71	100	100	1920 × 1080	Own
UAH DB (DS-2)	76	76	00	768 × 576	OA

**Table 2:** Class-wise distribution of the dataset used in this study.

Class		Real-Time (Own) (DS-1)	UAH DB (DS-2)
**Polyp**	**Hyperplastic**	60	21
	**Serrated**	30	15
	**Adenoma**	10	40
**Normal**		100	00
Total		200	76

**Table 3:** Dataset details after augmentation for training and validation.

Class	Real-Time (Own) (DS-1)		
	Original Dataset	Stage 1 Augmentation	Stage 2 Augmentation
**Hyperplastic**	60	60	1320
**Serrated**	30	60	1320
**Adenoma**	10	60	1320
**Normal**	100	60	1320
Total	200	240	5280

Note: Stage 1 data augmentation (S1DA): serrated data were augmented using 1 augmentation techniques’ (30 × 1 = 30) original data with S1DA (30 + 30 = 60); adenoma data were augmented using 5 augmentation techniques (10 × 5 = 50) on original data with S1DA (10 + 50 = 60); Stage 2 data augmentation (S2DA): used 11 augmentation techniques (60 × 21 = 1260) on original data with S1DA (60 + 1260 = 1320).

**Table 4:** Data split.

Class	Training (80%)	Validation (20%)	Testing (100%)	
	Real-Time (OWN) (DS-1)		Real-Time (Own) (DS-1)	UAH DB (DS-2)
**Hyperplastic**	1056	264	00	21
**Serrated**	1056	264	00	15
**Adenoma**	1056	264	00	40
**Normal**	1056	264	40	00
Total	4224	1056	40 + 76 = 116	

Note: After the Stage 2 data augmentation (S2DA): 80% of data (1056 images in each class) and 20% of data (264 images in each class) were used for training and validation, respectively.

**Table 5:** Classification results achieved using the VGG16 architecture.

Optimizers	Epoch	Class	Training						Validation					
			Accuracy	Sensitivity	Specificity	Precision	NPV	Overall Accuracy	Accuracy	Sensitivity	Specificity	Precision	NPV	Overall Accuracy
SGD	50/50	0	85.12	73.41	91.57	73.2	90.82	68.99	71.86	71.86	71.59	71.59	71.86	67.71
		1	84	68.99	90.62	72.06	88.92		64.39	64.39	67.80	67.80	64.39	
		2	83.92	70.48	90.27	71.69	89.42		64.47	64.47	66.67	66.67	64.47	
		3	85.99	73.24	91.11	68.94	90.85		70.66	70.66	64.77	64.77	70.66	
**ADAM**	**48/50**	**0**	**90.61**	**83.93**	**94.78**	**85.04**	**94.03**	**79.19**	**79.93**	**79.93**	**82.95**	**82.95**	**79.93**	**77.84**
		**1**	**89.91**	**80.26**	**93.85**	**82.77**	**92.94**		**76.03**	**76.03**	**76.89**	**76.89**	**76.03**	
		**2**	**89.24**	**81.55**	**92.92**	**81.63**	**92.37**		**76.14**	**76.14**	**76.14**	**76.14**	**76.14**	
		**3**	**91.29**	**84.33**	**95.16**	**80.49**	**94.5**		**79.28**	**79.28**	**75.38**	**75.38**	**79.28**	
RMSprop	50/50	0	88.85	80.73	93.41	81.72	92.59	75.19	78.08	78.08	76.89	76.89	78.08	73.96
		1	88.12	78.29	92.7	80.59	91.58		71.12	71.12	74.62	74.62	71.12	
		2	89.23	78.5	93.36	79.17	92.6		71.06	71.06	73.48	73.48	71.06	
		3	89.83	81.36	94.08	77.27	93.21		76.02	76.02	70.83	70.83	76.02	

Note: Class 0—normal, Class 1—hyperplastic, Class 2—serrated, and Class 3—adenoma. Optimal performance metrics are highlighted in bold, and all metrics are presented as percentages.

**Table 6:** Classification results achieved using the VGG19 architecture.

Optimizers	Epoch	Class	Training						Validation					
			Accuracy	Sensitivity	Specificity	Precision	NPV	Overall Accuracy	Accuracy	Sensitivity	Specificity	Precision	NPV	Overall Accuracy
SGD	50/50	0	85.12	73.41	91.57	73.2	90.82	71.47	75.49	75.49	72.35	72.35	75.49	70.08
		1	84	68.99	90.62	72.06	88.92		65.38	65.38	70.83	70.83	65.38	
		2	83.92	70.48	90.27	71.69	89.42		66.06	66.06	69.32	69.32	66.06	
		3	85.99	73.24	91.11	68.94	90.85		74.58	74.58	67.80	67.80	74.58	
**ADAM**	**47/50**	**0**	**90.61**	**83.93**	**94.78**	**85.04**	**94.03**	**82.48**	**84.53**	**84.53**	**84.85**	**84.85**	**84.53**	**80.87**
		**1**	**89.91**	**80.26**	**93.85**	**82.77**	**92.94**		**77.34**	**77.34**	**81.44**	**81.44**	**77.34**	
		**2**	**89.24**	**81.55**	**92.92**	**81.63**	**92.37**		**78.28**	**78.28**	**79.17**	**79.17**	**78.28**	
		**3**	**91.29**	**84.33**	**95.16**	**80.49**	**94.5**		**83.74**	**83.74**	**78.03**	**78.03**	**83.74**	
RMSprop	50/50	0	88.85	80.73	93.41	81.72	92.59	79.69	83.46	83.46	82.20	82.20	83.46	78.13
		1	88.12	78.29	92.7	80.59	91.58		73.59	73.59	79.17	79.17	73.59	
		2	89.23	78.5	93.36	79.17	92.6		74.44	74.44	76.14	76.14	74.44	
		3	89.83	81.36	94.08	77.27	93.21		81.82	81.82	75.00	75.00	81.82	

Note: Class 0—normal, Class 1—hyperplastic, Class 2—serrated, and Class 3—adenoma. Optimal performance metrics are highlighted in bold, and all metrics are presented as percentages.

**Table 7:** Classification results achieved using the ResNet50 architecture.

Optimizers	Epoch	Class	Training						Validation					
			Accuracy	Sensitivity	Specificity	Precision	NPV	Overall Accuracy	Accuracy	Sensitivity	Specificity	Precision	NPV	Overall Accuracy
SGD	46/50	0	90.12	84.38	95.16	84.94	94.45	83.76	87.80	87.80	84.47	84.47	87.80	81.91
		1	89.77	82.2	94.51	84.38	93.61		76.84	76.84	82.95	82.95	76.84	
		2	90.67	82.31	95.55	83.71	94.18		77.74	77.74	80.68	80.68	77.74	
		3	91.25	86.34	96.12	82.01	95.1		86.42	86.42	79.55	79.55	86.42	
**ADAM**	**39/50**	**0**	**95.12**	**91.04**	**97.55**	**90.44**	**96.65**	**89.2**	**90.26**	**90.26**	**91.29**	**91.29**	**90.26**	**88.07**
		**1**	**94.67**	**87.2**	**96.88**	**89.68**	**95.45**		**85.71**	**85.71**	**88.64**	**88.64**	**85.71**	
		**2**	**94.94**	**87.53**	**97.2**	**89.11**	**96.1**		**86.42**	**86.42**	**86.74**	**86.74**	**86.42**	
		**3**	**95.78**	**91.22**	**97.88**	**87.59**	**97.02**		**90.04**	**90.04**	**85.61**	**85.61**	**90.04**	
RMSprop	43/50	0	94.22	88.36	96.55	89.11	95.49	87.38	87.73	87.73	89.39	89.39	87.73	86.93
		1	93.69	85.87	96.13	88.07	94.69		85.24	85.24	87.50	87.50	85.24	
		2	94.06	86.37	96.47	87.03	95.14		85.77	85.77	86.74	86.74	85.77	
		3	95.02	89.03	97.12	85.32	96.43		89.16	89.16	84.09	84.09	89.16	

Note: Class 0—normal, Class 1—hyperplastic, Class 2—serrated, and Class 3—adenoma. Optimal performance metrics are highlighted in bold, and all metrics are presented as percentages.

**Table 8:** Classification results achieved using the DenseNet121 architecture.

Optimizers	Epoch	Class	Training						Validation					
			Accuracy	Sensitivity	Specificity	Precision	NPV	Overall Accuracy	Accuracy	Sensitivity	Specificity	Precision	NPV	Overall Accuracy
SGD	50/50	0	94.39	84.2	98.54	82.77	98.51	81.87	93.56	84.47	95.98	84.47	93.56	80.21
		1	93.73	79.61	98.52	82.1	98.25		91.48	78.14	94.92	82.58	91.48	
		2	93.81	79.87	98.51	81.53	98.26		89.96	77.53	93.56	78.41	89.96	
		3	94.42	84	98.31	81.06	98.33		90.53	80.89	92.67	75.38	90.53	
**ADAM**	**46/50**	**0**	**95.06**	**88.36**	**98.51**	**88.45**	**98.52**	**87.55**	**95.91**	**87.64**	**96.79**	**88.64**	**95.91**	**86.27**
		**1**	**94.82**	**86.66**	**98.48**	**87.97**	**98.32**		**94.36**	**85.45**	**95.83**	**86.74**	**94.36**	
		**2**	**94.84**	**86.75**	**98.49**	**87.41**	**98.31**		**93.84**	**85.17**	**95.72**	**84.85**	**93.84**	
		**3**	**95.09**	**88.46**	**98.34**	**86.36**	**98.33**		**94.41**	**86.82**	**95.72**	**84.85**	**94.41**	
RMSprop	50/50	0	93.12	87.46	98.79	87.46	93.12	86.27	94.74	85.87	96.55	87.5	94.74	84.66
		1	92.73	84.66	98.66	86.74	92.55		93.37	82.91	95.83	86.36	93.37	
		2	92.64	85.22	98.53	86.27	92.37		92.84	83.77	95.08	84.09	92.84	
		3	93.14	87.84	98.61	84.85	93.18		93.66	86.23	94.66	80.68	93.66	

Note: Class 0—normal, Class 1—hyperplastic, Class 2—serrated, and Class 3—adenoma. Optimal performance metrics are highlighted in bold, and all metrics are presented as percentages.

**Table 9:** Classification results achieved using the EfficientNetV2 architecture.

Optimizers	Epoch	Class	Training						Validation					
			Accuracy	Sensitivity	Specificity	Precision	NPV	Overall Accuracy	Accuracy	Sensitivity	Specificity	Precision	NPV	Overall Accuracy
SGD	47/50	0	91.37	83.38	94.83	84.56	94.38	83.07	92.8	85.82	94.95	84.85	95.01	81.06
		1	90.73	81.48	94.66	84.19	93.63		89.2	77.03	94.05	82.58	91.82	
		2	90.86	81.25	94.13	82.48	93.67		89.2	77.37	93.37	80.3	92.16	
		3	92.02	86.46	93.81	81.06	95.77		92.8	84.87	95.6	76.52	95.6	
**ADAM**	**43/50**	**0**	**94.42**	**90.24**	**96.44**	**89.3**	**96.44**	**88.66**	**94.92**	**89.43**	**96.59**	**89.77**	**96.47**	**87.69**
		**1**	**93.97**	**87.27**	**96.3**	**88.92**	**95.68**		**93.3**	**85.04**	**96.03**	**88.26**	**94.81**	
		**2**	**94.04**	**87.45**	**96.13**	**88.45**	**95.77**		**93.94**	**86.14**	**95.69**	**87.12**	**95.33**	
		**3**	**94.97**	**89.76**	**96.65**	**87.97**	**96.65**		**95.45**	**90.4**	**97.04**	**85.61**	**97.06**	
RMSprop	45/50	0	93.91	88.67	96.04	88.16	96.06	86.98	94.01	86.89	95.97	87.88	95.94	85.70
		1	92.26	85.45	95.92	87.88	95		92.8	84.13	95.42	86.36	94.57	
		2	92.84	85.67	95.52	86.65	95.17		93.08	84.7	95.29	85.98	94.82	
		3	94.27	88.24	96.26	85.23	96.26		93.94	87.2	96.01	82.58	96.01	

Note: Class 0—normal, Class 1—hyperplastic, Class 2—serrated, and Class 3—adenoma. Optimal performance metrics are highlighted in bold, and all metrics are presented as percentages.

**Table 10:** Classification results achieved using the InceptionNetV3 architecture.

Optimizers	Epoch	Class	Training						Validation					
			Accuracy	Sensitivity	Specificity	Precision	NPV	Overall Accuracy	Accuracy	Sensitivity	Specificity	Precision	NPV	Overall Accuracy
SGD	49/50	0	92.12	83.62	94.56	83.62	94.57	82.55	92.71	84.85	98.75	84.85	92.71	80.59
		1	91.1	82.77	94.2	82.77	93.29		91.43	78.7	98.56	82.58	91.05	
		2	91.3	82.29	94.1	82.29	93.46		90.73	77.7	98.14	79.17	90.36	
		3	92.61	81.53	94.86	81.53	95.19		91.96	81.3	98.08	75.76	92.33	
**ADAM**	**45/50**	**0**	**96.61**	**89.07**	**98.74**	**88.75**	**98.74**	**88.04**	**96.15**	**87.55**	**98.9**	**87.88**	**98.89**	**86.62**
		**1**	**96.47**	**86.88**	**98.95**	**88.45**	**98.64**		**95.84**	**85.33**	**98.92**	**87.03**	**98.75**	
		**2**	**96.5**	**86.96**	**98.97**	**87.78**	**98.65**		**95.92**	**85.61**	**98.93**	**86.17**	**98.74**	
		**3**	**96.8**	**89.33**	**98.85**	**87.22**	**98.87**		**96.3**	**88.09**	**98.76**	**85.42**	**98.77**	
RMSprop	48/50	0	96.15	87.55	98.9	87.88	98.89	86.62	92.9	86.25	98.56	87.88	92.51	84.94
		1	95.84	85.33	98.92	87.03	98.75		91.82	83.15	98.18	85.98	91.34	
		2	95.92	85.61	98.93	86.17	98.74		91.75	84.03	97.99	83.71	91.26	
		3	96.3	88.09	98.76	85.42	98.77		92.79	86.45	98.15	82.2	92.45	

Note: Class 0—normal, Class 1—hyperplastic, Class 2—serrated, and Class 3—adenoma. Optimal performance metrics are highlighted in bold, and all metrics are presented as percentages.

**Table 11:** Classification results achieved using the ViT architecture.

Optimizers	Epoch	Class	Training						Validation					
			Accuracy	Sensitivity	Specificity	Precision	NPV	Overall Accuracy	Accuracy	Sensitivity	Specificity	Precision	NPV	Overall Accuracy
SGD	42/50	0	93.45	87.05	95.82	87.88	94.95	86.36	85.77	85.77	86.74	86.74	85.77	84.85
		1	92.49	84.73	95.36	87.22	93.91		84.27	84.27	85.23	85.23	84.27	
		2	93	85.51	95.6	86.08	94.28		83.77	83.77	84.09	84.09	83.77	
		3	93.85	88.29	96.18	84.28	95.25		85.60	85.60	83.33	83.33	85.60	
**ADAM**	**35/50**	**0**	**96.36**	**93.45**	**97.92**	**93.18**	**97.21**	**92.38**	**96.4**	**91.85**	**97.96**	**93.94**	**97.22**	**91.1**
		**1**	**95.71**	**91.47**	**97.85**	**91.38**	**96.29**		**95.27**	**89.93**	**97.08**	**91.29**	**96.59**	
		**2**	**95.8**	**91.5**	**97.89**	**92.71**	**96.08**		**95.17**	**90.49**	**96.72**	**90.15**	**96.84**	
		**3**	**96.35**	**93.12**	**98.17**	**92.23**	**96.92**		**95.36**	**92.16**	**96.38**	**89.02**	**97.47**	
RMSprop	39/50	0	94.53	91.11	96.74	91.19	95.89	90.34	95.27	88.93	97.96	91.29	96.59	89.11
		1	93.84	89.81	96.68	90.15	95.48		94.21	87.82	97.08	90.15	96.84	
		2	93.7	89.55	96.55	89.3	95.25		94.07	87.92	96.72	88.26	97.22	
		3	94.1	90.89	96.86	90.72	95.74		94.4	91.97	96.38	86.74	97.47	

Note: Class 0—normal, Class 1—hyperplastic, Class 2—serrated, and Class 3—adenoma. Optimal performance metrics are highlighted in bold, and all metrics are presented as percentages.

**Table 12:** Classification results achieved using CRP-ViT (multi-class) architecture.

Optimizers	Epoch	Class	Training						Validation					
			Accuracy	Sensitivity	Specificity	Precision	NPV	Overall Accuracy	Accuracy	Sensitivity	Specificity	Precision	NPV	Overall Accuracy
SGD	35/50	0	92.52	92.43	97.22	91.19	95.7	91.76	95.51	91.63	97.94	91.29	96.86	89.77
		1	91.04	91.04	96.67	91.38	95.31		93.76	86.86	97.08	90.15	96.41	
		2	91.11	91.11	96.44	91.19	95.1		94.26	88.72	96.7	89.39	97.12	
		3	92.48	92.48	97.28	91.95	95.83		94.88	92.09	96.45	88.26	97.54	
**ADAM**	**22/50**	**0**	**97.92**	**97.55**	**99.31**	**97.44**	**98.9**	**97.28**	**98.01**	**97.75**	**98.29**	**98.86**	**98.51**	**96.02**
		**1**	**96.44**	**96.44**	**99.13**	**97.25**	**98.5**		**95.93**	**93.8**	**97.97**	**97.35**	**96.2**	
		**2**	**97.16**	**97.16**	**99.15**	**96.5**	**98.75**		**96.57**	**95.08**	**96.68**	**95.08**	**97.04**	
		**3**	**97.98**	**97.98**	**99.43**	**97.98**	**99**		**96.97**	**97.61**	**96.8**	**92.8**	**99.02**	
RMSprop	29/50	0	96.49	96.49	98.92	96.21	98.6	95.45	97.35	95.54	98.8	97.35	98.65	94.22
		1	94.39	94.39	98.75	95.27	98.2		93.94	92.54	95.1	93.94	96.15	
		2	94.82	94.82	98.6	94.79	98.3		92.8	93.51	92.7	92.8	96.1	
		3	96.16	96.16	99.01	94.79	98.5		92.8	95.33	91.9	92.8	99.1	

Note: Class 0—normal, Class 1—hyperplastic, Class 2—serrated, and Class 3—adenoma. Optimal performance metrics are highlighted in bold, and all metrics are presented as percentages.

**Table 13:** K-fold cross validation on CRP-ViT (multi-class).

K-Fold	Class	Accuracy	Sensitivity	Specificity	Precision	NPV	Overall Accuracy
K = 1	0	99.15	97.75	99.62	98.86	99.24	96.69
	1	98.11	94.85	99.23	97.73	98.23	
	2	98.01	96.2	98.61	95.83	98.74	
	3	98.11	98.03	98.13	94.32	99.37	
K = 2	0	99.23	97.77	99.62	99.62	99.24	97.92
	1	98.49	97.37	99.04	98.11	98.49	
	2	98.2	97.36	98.86	97.73	99.24	
	3	98.77	99.22	98.7	96.21	99.79	
K = 3	0	97.11	96.27	97.73	97.73	97.12	95.08
	1	95.9	93.68	97.04	95.45	96.56	
	2	95.52	94.68	95.84	94.32	96.83	
	3	94.87	95.7	94.74	92.8	98.16	
K = 4	0	97.11	95.56	97.73	96.21	97.12	95.17
	1	95.9	94.78	96.21	93.56	96.56	
	2	95.52	94.64	95.84	93.18	96.83	
	3	94.87	95.72	94.74	93.18	98.16	
K = 5	0	98.48	97.01	98.48	96.97	98.24	96.5
	1	96.97	95.52	96.97	96.59	97.25	
	2	96.59	95.15	96.59	93.94	98.05	
	3	95.83	98.41	95.83	93.94	99.48	
Mean ± SD	0	98.22 ± 0.94	96.87 ± 0.86	98.64 ± 0.85	97.88 ± 1.24	98.19 ± 0.95	96.27 ± 1.06
	1	97.07 ± 1.02	95.24 ± 1.22	97.70 ± 1.21	96.29 ± 1.65	97.42 ± 0.81	
	2	96.77 ± 1.06	95.61 ± 1.04	97.15 ± 1.33	95.00 ± 1.61	97.94 ± 0.98	
	3	96.89 ± 1.55	97.42 ± 1.45	96.43 ± 1.68	94.09 ± 1.19	98.99 ± 0.69	

Note: Class 0—normal, Class 1—hyperplastic, Class 2—serrated, and Class 3—adenoma., and all metrics are presented as percentages.

**Table 14:** Difference between 80:20 and K-fold.

Method	Class	Accuracy	Sensitivity	Specificity	Precision	NPV	Overall Accuracy
80:20 Split (Hold-Out) (A)	0	98.01	97.75	98.29	98.86	98.51	96.02
	1	95.93	93.8	97.97	97.35	96.2	
	2	96.57	95.08	96.68	95.08	97.04	
	3	96.97	97.61	96.8	92.8	99.02	
K-Fold Cross (Leave-Out) (B)	0	98.22	96.87	98.64	97.88	98.19	96.27
	1	97.07	95.24	97.7	96.29	97.42	
	2	96.77	95.61	97.15	95	97.94	
	3	96.49	97.42	96.43	94.09	98.99	
Difference Between (A and B)	0	0.21	0.88	0.35	0.98	0.32	0.25
	1	1.14	1.44	0.27	1.06	1.22	
	2	0.2	0.53	0.47	0.08	0.9	
	3	0.48	0.19	0.37	1.29	0.03	

Note: Class 0—normal, Class 1—hyperplastic, Class 2—serrated, and Class 3—adenoma; all metrics are presented as percentages.

**Table 15:** Ablation study for CRP-ViT (multi-class) architecture.

Removal/Modification Techniques Applied to ResNet50 Architecture Techniques	Accuracy
Stage 1 layer modification	90.18
Stage 2 layer modification	91.71
Stage 3 layer modification	92.78
Stage 4 layer modification	90.74
Simultaneous modifications across Stages 1 to 4	67.06
Modification at the fully connected (FC) layer	87.85
**CRP-ViT**	**97.28**

Note: Optimal performance metrics are highlighted in bold, and all metrics are presented as percentages.

## Data Availability

The data that support the findings of this study are openly available at the following URL: http://www.depeca.uah.es/colonoscopy_dataset/; (accessed on 18 April 2025) and DOI: https://doi.org/10.1109/tmi.2016.2547947.

## Abbreviations

The following abbreviations are used in this manuscript:

CAD	Computer-Aided Diagnosis
CNN	Convolutional Neural Network
CRC	Colorectal Cancer
CRP	Colorectal Polyp
CT	Computed Tomography
D&T	Diagnostic and Therapeutic
DA	Domain Adaptation
DA	Data Augmentation
DL	Deep Learning
DNA	Deoxyribonucleic Acid
DS	Dataset
DS-1	Dataset 1 (Real-Time (own))
DS-2	Dataset 2 (UAH DB)
GUI	Graphical User Interface
IBD	Inflammatory Bowel Disease
IEC	Institutional Ethics Committee
ML	Machine Learning
MLP	Multi-Layer Perceptron
ROC	Receiver Operating Characteristic
S1DA	Stage 1 Data Augmentation
S2DA	Stage 2 Data Augmentation
SSP	Sessile Serrated Polyps
TSA	Traditional Serrated Adenomas
ViT	Vision Transformer
WCE	Wireless Capsule Endoscopy
